# Hospital Construction Competitions

**Published:** 1912-04-27

**Authors:** Edwin T. Hall

**Affiliations:** The Assessor. 54 Bedford Square, London, W.C.


### THE EDITOR'S LETTER-BOX.

### Hospital Construction Competitions.

THE EAST SUSSEX HOSPITAL, HASTINGS.

Sir,—My attention was drawn to your issue of March 2, in which, on pages 563-4-5, you deal with competitions in general, and that of the East Sussex Hospital in particular, and I have read the correspondence that followed in more recent numbers of your paper.

I have been expecting that the mis-statements by your anonymous contributors (each of whom you state is a Fellow of the R.I.B.A.) of (1) the special report, and (2) the note—to whom, for brevity, I shall hereafter refer as "No. 1" and "No. 2"—would have been corrected, and apologies offered, especially after Mr. A. Saxon Snell had pointed out the inaccuracy in the statement as to the condition regarding cost, and had suggested that the names of both contributors should be given.

They, apparently, have not the courage to give their names.

I have now the duty of setting out the points to which I take exception.

(1) "No. 1" quotes largely from the Instructions to Architects, and then concludes : "The impression left in our (*sic*) mind after reading the schedule and the questions by competitors with the assessor's replies is that the whole scheme has been carelessly drawn up, and shows in many instances a lamentable lack of knowledge on the part of their adviser." The word "adviser" can only refer to me. I demand a withdrawal of the statements and an apology.

(2) "No. 1" states : "The committee fixed the sum of £35,000 . . . . *as the total cost* . . . . "; and again he says, "the amount *fixed as a maximum* . . . . "; and "No. 2" says, "*the cost was stipulated as being an essential in the selection*" . . . ; and, again, "the selected design is about £7,000 *above the sum fixed as essential in the selection.*" The italics are mine. These statements are the baseless fabric of your contributors' imagination, and your editorial preface, founded on such evidence, says : "When a committee instructs the assessor to draw his conditions to include a hospital of a given number of beds not to exceed the maximum sum, such assessor is bound by his instructions and should disqualify all competitors who submit plans, the estimated cost of which exceeds the amount fixed for the guidance of every competitor." Your comment is either irrelevant to this competition or it is a reflection on me.

The actual Condition No. 12 is as follows :

"The committee think that £35,000 is the maximum that they will have at their disposal for the whole hospital, and while they require the hospital to be up-to-date in all particulars, preference will be given to a design within that sum, if possible, but competitors must use their discretion in designing if they think that amount insufficient." There is not one word in the condition about fixing £35,000 as the total or maximum cost, or that £35,000 was "the sum fixed as essential in the selection." Again, in clause 28, which states the grounds on which "designs will be excluded," or to use your own word "disqualified," there is not one word to say that if the cost of any design exceeds £35,000 it will be excluded.

For these mis-statements of "Nos. 1 and 2," and your prefatory comment, I must again ask for a withdrawal and apology.

(3) "No. 2" states: "The assessor's conditions show a lack of knowledge both of the site and the application of the conditions thereto; it being assumed that the site was level." With the conditions a plan of the site was sent to each competitor, giving the levels all over, and "No. 1" sets out, no doubt from the plan, the differences in levels as sixty feet from west to east. Condition No. 26 further points out that "Replies" to any questions by competitors "shall be read as forming part of these conditions."

Question 3 shows a diagram of steeply sloping ground, and asks if a room above the lower ground, but nearly below the upper, would be considered in the basement, and an answer is given. Question 11 asks: "Will an arrangement suiting the fall of the land by making the women's pavilion three storeys high and placing the children's ward in the lower floor be considered?" and the answer is "Yes, if all above ground." Questions and Answers 32 and 52 also relate to levels of different parts of the site.

"No. 2's" statement is, therefore, shown to be without foundation, and I ask for its withdrawal and for an apology.

I have to acknowledge the courtesy and propriety of Messrs. P. Adams and Holden, who were placed third, in disclaiming any connection with the aspersions made, and also to thank Mr. A. Saxon Snell for his letter.

I would ask that you give the same prominence and publicity to this letter as you gave to the original articles.

I am, Sir, your obedient servant,

Edwin T. Hall, F.R.I.B.A.,

The Assessor.

54 Bedford Square, London, W.C.,
April 15.

